# Role of Toll-Like Receptor 4 in Mycobacterium avium subsp. *paratuberculosis* Infection of Bovine Mammary Epithelial (MAC-T) Cells *In Vitro*

**DOI:** 10.1128/spectrum.04393-22

**Published:** 2023-03-13

**Authors:** Umesh K. Shandilya, Xiang Wu, Caitlin McAllister, Lucy Mutharia, Niel A. Karrow

**Affiliations:** a Department of Animal Biosciences, University of Guelph, Guelph, Ontario, Canada; b Department of Molecular and Cellular Biology, University of Guelph, Guelph, Ontario, Canada; University of North Dakota

**Keywords:** Toll-like receptor 4 (TLR4), bovine mammary epithelial (MAC-T) knockout cells, *Mycobacterium avium* subsp. *paratuberculosis* (MAP), miRNA, cytokines

## Abstract

Toll-like receptor 4 (*TLR4*) encodes an innate immune cell pattern-recognition receptor implicated in the recognition of Mycobacterium avium subsp. *paratuberculosis* (MAP), the causative agent of Johne’s disease in ruminants. Polymorphisms in *TLR4* have been associated with susceptibility to MAP infection. In this study, a previously developed *TLR4* knockout (*TLR4*KO) bovine mammary epithelial (MAC-T) cell line and wild-type MAC-T cells (WT) were infected with live MAP for 72 h to identify potential immunoregulatory miRNAs, inflammatory genes, and cytokines/chemokines impacted by MAP infection in the presence/absence of *TLR4*. Cytokines/chemokines production in culture supernatants was measured by multiplexing immunoassay. Total RNA was extracted from the remaining MAC-T cells, and quantitative PCR was performed to determine the expression of inflammatory genes and selected bovine miRNAs. Results showed that the levels of tumor necrosis factor-α (TNF-α), interleukin-6 (IL-6), CXCL8, CXCL10, CCL4, and CCL3 were significantly induced in WT MAC-T cells during MAP infection. However, *TLR4*KO MAC-T cells had greater secretion of CCL3, IL-6, vascular endothelial growth factor (VEGF-α), and TNF-α and decreased secretion of CXCL10 and CCL2. Moreover, the expression of inflammatory genes was induced in *TLR4*KO cells. The expression of miRNAs (miR133b, miR-92a, and miR-184) was increased in WT MAC-T cells post-MAP infection; however, there was no significant induction of these miRNAs in *TLR4*KO cells, which suggests they are involved in regulating the innate immune response to MAP infection. Target gene function analysis further suggests that miR-92a may be involved in TLR and interleukin signaling and miR-133b and miR-184 may be involved in other signaling pathways. These findings support the involvement of TLR4 in the regulation of innate immune response to MAP.

**IMPORTANCE** Mycobacterium avium subsp. *paratuberculosis* (MAP) is the causative agent for paratuberculosis or Johne’s disease (JD) in ruminants, a disease clinically very similar to Crohn’s disease in humans. Polymorphisms in the bovine Toll-like receptor genes (*TLR1*, *TLR2*, and *TLR4*) have been shown to affect MAP recognition and host innate immune response and have been associated with increased susceptibility of cattle to paratuberculosis. Our results demonstrated that knocking out the *TLR4* gene in bovine MAC-T cells enhanced inflammation in response to MAP. These findings show divergent roles for TLR4 in Escherichia coli lipopolysaccharide and mycobacterial infections, and this may have important consequences for the treatment of these inflammatory diseases and for genetic selection to improve disease resistance. It advances our understanding of the role of TLR4 in the context of MAP infection.

## INTRODUCTION

Johne’s disease (JD), also known as paratuberculosis, is a chronic progressive intestinal inflammatory disease of cattle caused by infection with Mycobacterium avium subsp. *paratuberculosis* (MAP). MAP infection leads to intestinal damage in cattle, which can affect nutrient absorption that results in a decrease in milk yield and early culling. MAP is zoonotic, and it has long been considered to be a potential cause of Crohn’s disease (1). Cattle with subclinical MAP infection can intermittently or persistently shed MAP in their feces (2) and also milk (3), which increases the risk of exposure to herd mates, farm operators, and consumers. Currently, there is neither an effective treatment option nor a vaccine that can prevent MAP infection and provide protective immunity (4), which collectively makes controlling MAP a global challenge.

Polymorphisms in the bovine Toll-like receptor genes (*TLR1*, *TLR2,* and *TLR4*) have been associated with increased susceptibility of cattle to paratuberculosis (5–7) and have been shown to affect pathogen recognition and host innate immune response (8). Although TLR-ligand interactions have been well characterized, new ligands and TLR interactions are continually being discovered. Certain bacteria, the spirochetes, for example, are recognized by TLR2 as well as TLR4 (9), and heat shock protein (HSP), an alarmin released during tissue damage, was shown to be recognized by both TLR2 and TLR4 (10), which can synergize (11). Although TLR4 is mainly involved in bacterial lipopolysaccharide (LPS) recognition, it also recognizes a variety of host alarmins (12). Although TLR4 has been found to recognize ligands from other mycobacteria subspecies (13), the role of TLR4 in MAP recognition is less certain and warrants investigation.

Mammary epithelial cells (MECs) act as a defensive barrier against intramammary pathogens and help initiate an appropriate protective immune response against invading pathogens after their recognition by MEC pattern-recognition receptors such as TLR4. MEC culture models have been used to better understand host-pathogen interactions (14, 15), including MEC-MAP interactions (16, 17). Our previous study, for example, demonstrated that TLR4 plays a pivotal role in triggering inflammation induced by MAP cell lysate (8).

MAP has been demonstrated to evade the immune system, in part, by downregulating major histocompatibility complex (MHC) expression, which impairs antigen presentation thereby delaying T-cell recognition and the secretion of tumor necrosis factor-α (TNF-α) (18) and interferon-γ (IFN-γ) and expression, which are required for protective immunity (19). Cytokines are associated with protection from immunopathology following MAP infection (20) and may indeed play an important role in the immune responses to MAP infection and the development or control of JD (21).

MAP may also interfere with the expression of immunoregulatory microRNAs (miRNA). Some miRNAs play an important role in regulating innate immunity by targeting TLRs and/or proteins within the TLR signaling pathways (22, 23). Infection with M. tuberculosis increased miR-1178 expression in human macrophages, which could regulate the immune response of macrophages by targeting TLR4 (24). Niu et al. (25) demonstrated that miR-125a decreased macrophage inflammatory cytokine production during M. tuberculosis infection, and miR-125a levels were dramatically reduced by the knockdown of *TLR4* in THP-1 cells during M. tuberculosis infection. In addition, Li and Zhang (26) found that miR-708-5p influenced M. tuberculosis viability and the human macrophage inflammatory response during infection by targeting TLR4 and miR-708-5p mimics reduced proinflammatory factors including IFN-γ, interleukin-6 (IL-6), IL-1β, and TNF-α.

The exact mechanisms by which MAP immune evasion occurs are not completely understood. It is hypothesized that during the interaction between the host immune system and MAP, a deviation from a protective immune response arises, and this disrupts the ability of the host to contain the pathogen, which leads to its dissemination throughout the body. Therefore, this study aimed to characterize the functional roles of TLR4 in response to live MAP challenge.

## RESULTS

### Cytokine/chemokine production.

**FIG 1:**
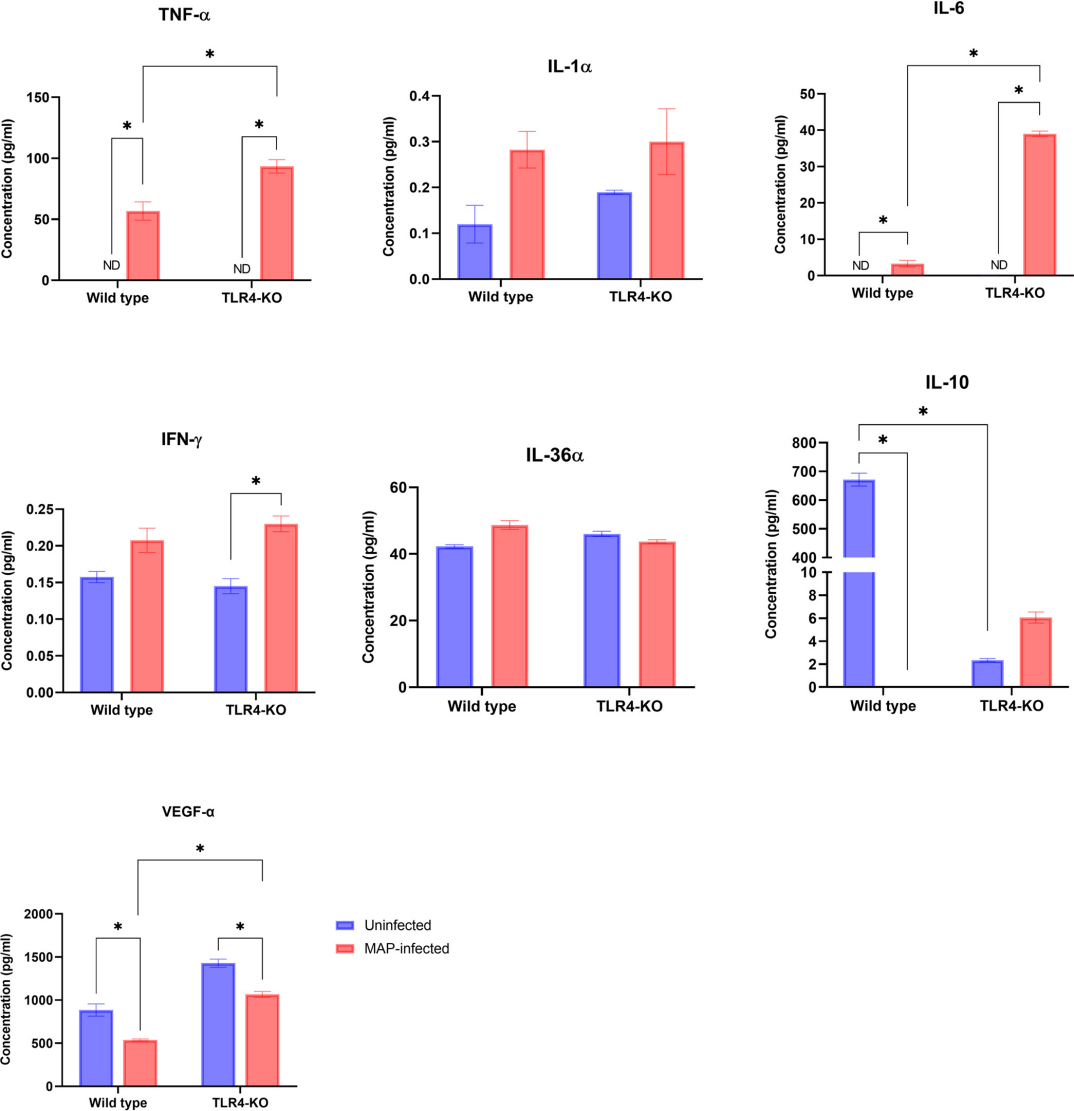
Cytokine concentrations from the culture supernatant of *TLR4* knockout (TLR4-KO) and wild-type bovine MAC-T cells challenged with Mycobacterium avium subsp. paratuberculosis (MAP) for 72 h. Data are expressed as mean (pg/mL) and SEM. Significant differences are denoted by *, *P* < 0.05; ND, not detected.

**FIG 2:**
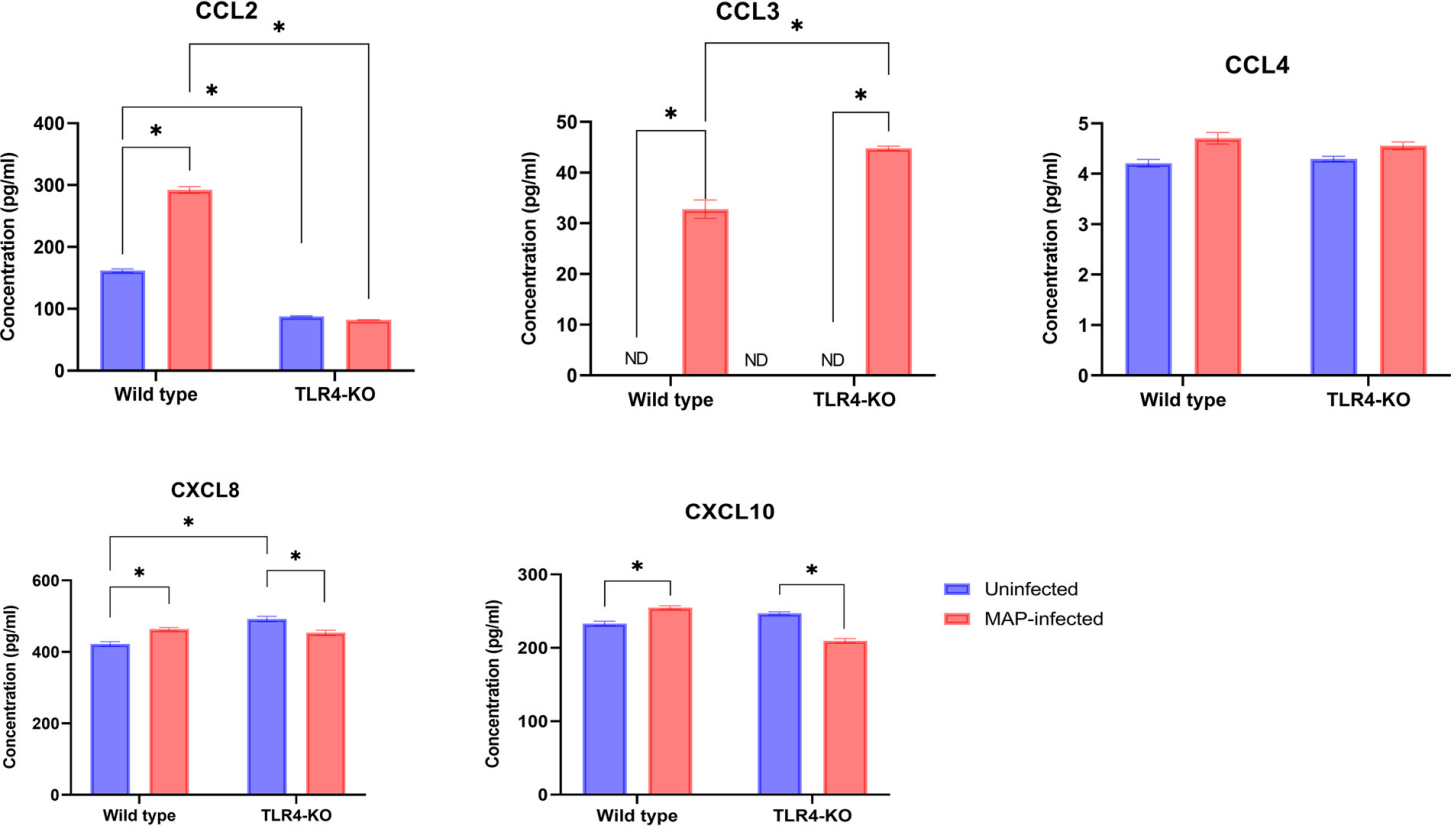
Chemokine concentrations in culture supernatant of *TLR4* knockout (TLR4-KO) and wild-type bovine MAC-T cells challenged with Mycobacterium avium subsp. paratuberculosis (MAP) for 72 h. Data are expressed as mean (pg/mL) and SEM. Significant differences are denoted by *, *P* < 0.05; ND, not detected.

The effect of MAP infection on cytokine/chemokine production by wild-type (WT) and *TLR4* knockout (*TLR4*KO) bovine mammary epithelial (MAC-T) cells was determined in the culture supernatants by multiplex analysis. The levels of TNF-α, IL-6, CXCL8, CXCL10, CCL2, and CCL3 were significantly induced in MAP-infected WT cells compared with uninfected WT cells (Fig. 1 and 2). Conversely, significantly smaller amounts of IL-10 and vascular endothelial growth factor (VEGF-α) were secreted by MAP-infected WT cells versus the uninfected WT cells. For the *TLR4*KO cells, the levels of TNF-α, IL-6, IFN-γ, and CCL3 were significantly induced (*P* < 0.05) in MAP-infected cells compared to corresponding uninfected *TLR4*KO cells, whereas, VEGF-α, CXCL10, and CXCL8 were significantly lower. However, secretion of IL-10, CCL4, IL-36α, CCL2, and IL-1α was not significantly different between the MAP-infected and uninfected *TLR4*KO cells. When comparing MAP-infected *TLR4*KO and WT cells, TNF-α, VEGF-α, IL-6, and CCL3 were secreted significantly more by the *TLR4*KO cells than WT cells, and CXCL10 and CCL2 were secreted significantly less by the *TLR4*KO cells than WT cells.

### Gene expression.

**FIG 3:**
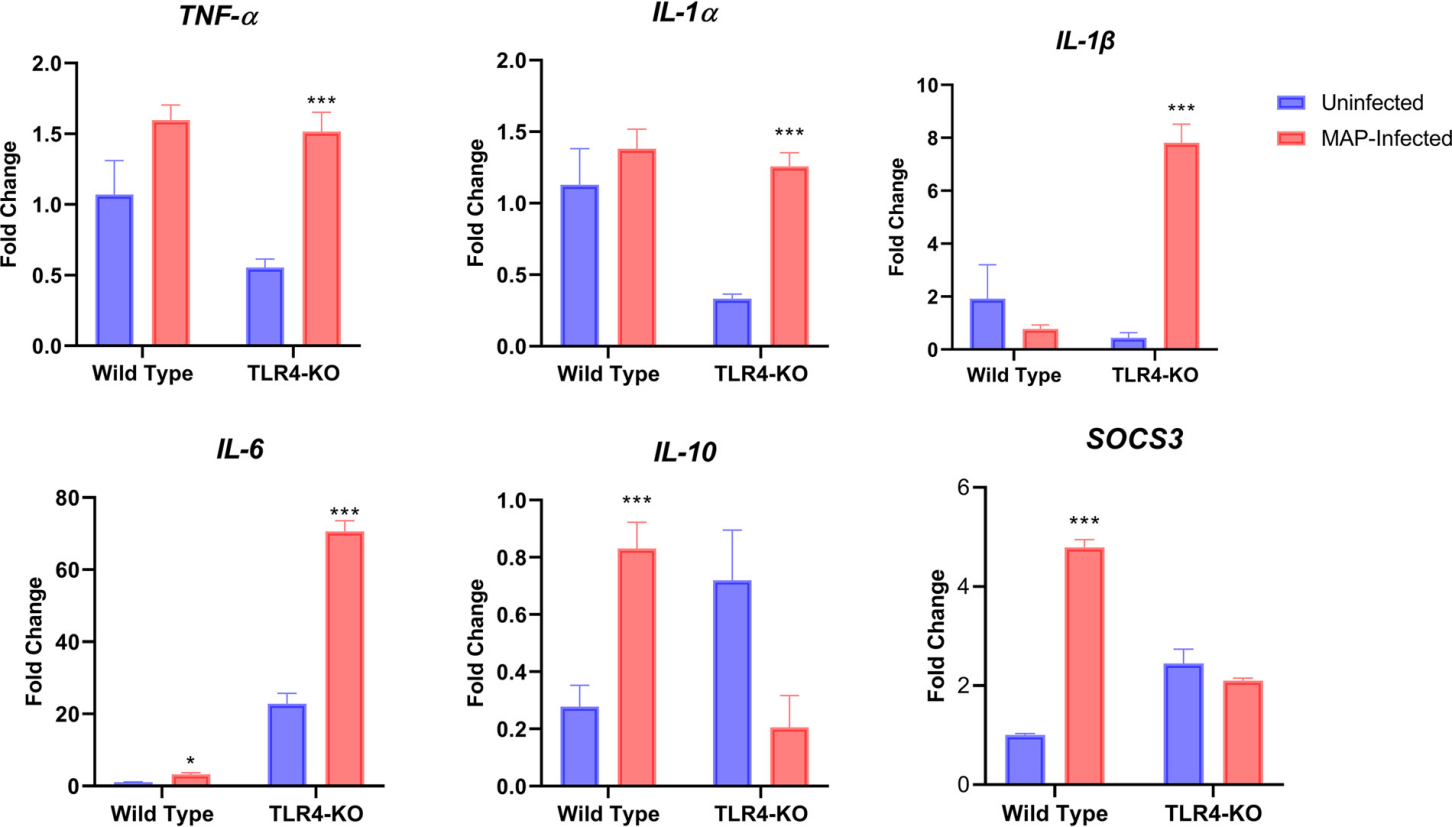
Relative fold change of proinflammatory (*TNF*-α, *IL-1*α, *IL-1*β and *IL-6*) and anti-inflammatory (*SOCS3* and *IL-10*) cytokine gene expression in *TLR4* knockout (TLR4-KO) and wild-type MAC-T cells challenged with Mycobacterium avium subsp. paratuberculosis (MAP) for 72 h. Data are expressed as mean ± SEM (*n* = 4). Significant differencesare denoted by *, *P* < 0.05 and ***, *P* < 0.001.

The expression of proinflammatory cytokine genes *TNF-α*, *IL-6*, *IL1α*, and *IL1β* was significantly increased in *TLR4*KO cells post-MAP infection for 72 h (*P* < 0.05) compared to corresponding uninfected *TLR4*KO cells, but no significant change was observed WT cells post-MAP infection (Fig. 3). On the other hand, expression of *IL-10*, an anti-inflammatory cytokine, showed a significant decrease (*P* < 0.05) in *TLR4*KO cells compared to corresponding uninfected cells, and the opposite response was observed for the WT cells, where *IL-10* expression was found to increase (*P* < 0.05). Expression of another anti-inflammatory cytokine, *SOCS3*, was also increased in WT cells during MAP infection, but no significant change in *SOCS3* expression was detected in *TLR4*KO cells.

### microRNA expression.

**FIG 4:**
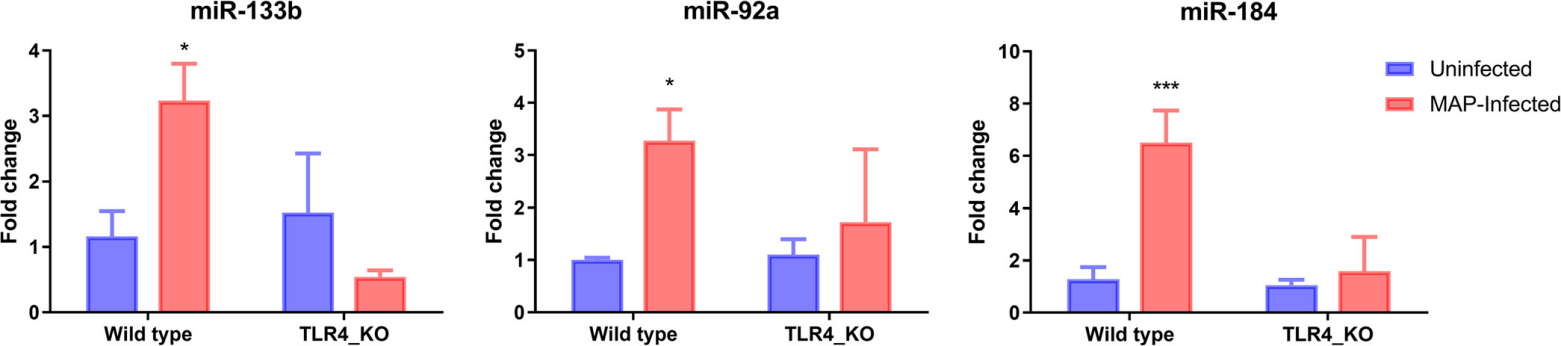
Differential expression of miRNAs in *TLR4* knockout (TLR4-KO) and wild-type MAC-T cells post-MAP-infection for 72 h. Data are expressed as mean ± SEM (*n* = 4). Significant differences are denoted by *, *P* < 0.05 and ***, *P* < 0.001.

In this study, we also analyzed the expression of three bovine miRNAs (miR-133b, miR-92a, and miR-184) at 72 h post-MAP infection. MAP-induced miRNA expression in MAC-T cells is shown in Fig. 4. We found that the WT cells challenged with live MAP had significant induction of expression of all three miRNAs compared to the uninfected WT cells (*P* < 0.05). However, the expression of these miRNAs was not significantly affected in *TLR4*KO MAC-T cells post-MAP infection compared to uninfected *TLR4*KO cells.

### Gene enrichment and pathways analysis.

**FIG 5:**
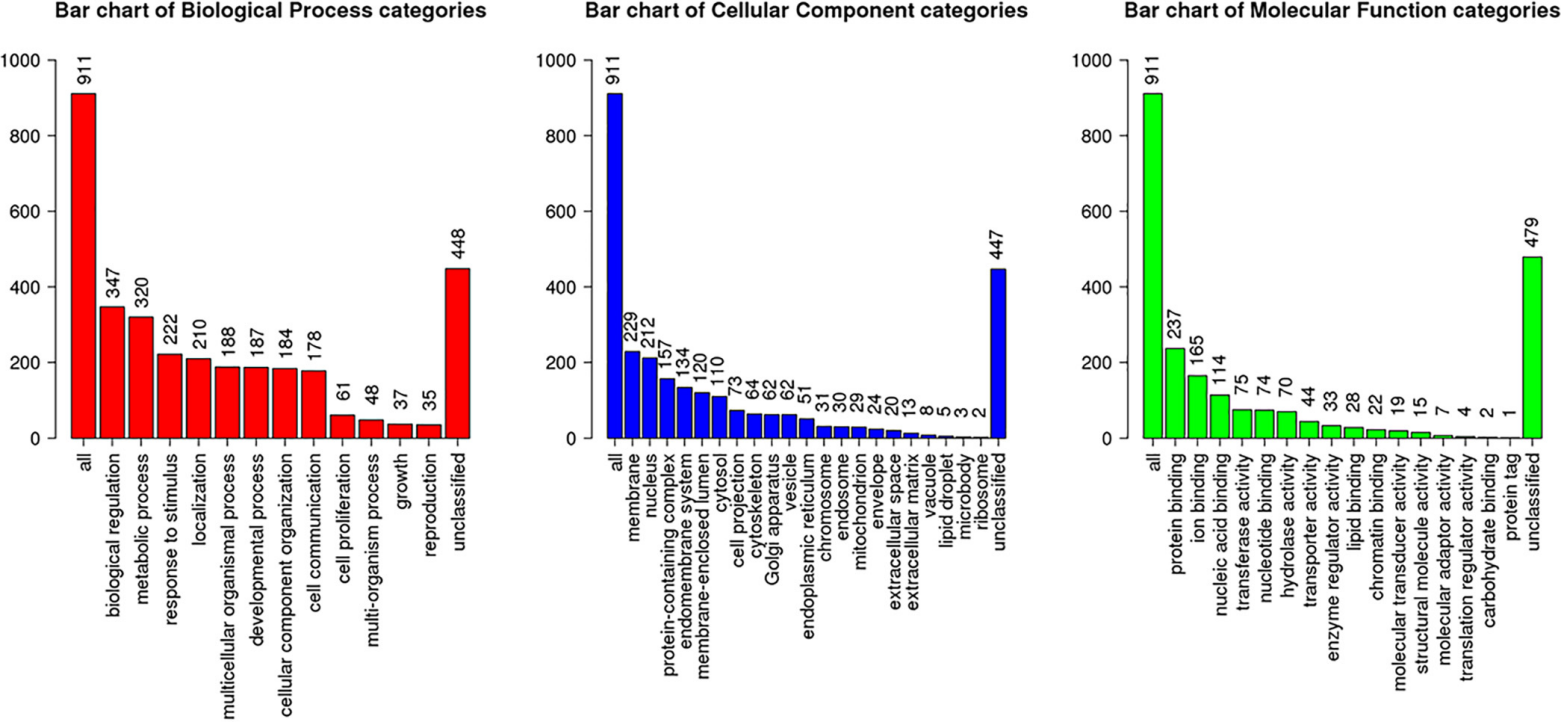
Bar chart of gene ontology (GO) terms predicted by identified target genes of upregulated bovine miRNAs (miR-133b, miR-92a, and miR-184) using Web-GSTALT online tool in three categories: biological processes, cellular components, and molecular functions.

A total of 1298 (miR-133b), 1932 (miR-92a), and 99 (miR-184) target genes were predicted by two different target gene prediction tools (miRDb and TargetScan). The commonly identified target genes were considered for functional analysis. Functional gene enrichment analysis of predicted target genes was then performed using the WeB-Gestalt webserver, as shown in Fig. 5. Bovine miR-133b and miR-92a were found to be involved in the inflammatory signaling pathways; however, miR-184 was found to be involved in the Notch signaling pathway as shown in Table 1. The top three enriched terms in the biological process categories were biological regulation, metabolic process, and response to stimulus. The top three enriched terms in the cellular component categories were membrane, nucleus, and protein-containing complex. The top three enriched terms in the molecular function categories were protein binding, ion binding, and nucleic acid binding.

**TABLE 1:** Numbers of commonly identified target genes of miR-133b, miR-92a, and miR-184 and selected pathways

miRNA	TargetScan	miRDB	Common targets	Selected pathways
miR-133b	644	654	352	p38 MAPK pathway; Notch signaling pathway; PI3 kinase pathway; Wnt signaling pathway; Inflammation mediated by chemokine and cytokine signaling pathway.
miR-92a	947	985	612	Interleukin signaling pathway; Toll receptor signaling pathway; Inflammation mediated by chemokine and cytokine signaling pathway.
miR-184	29	70	11	Notch signaling pathway.

## DISCUSSION

The immune response to MAP infection in cattle involves a complex interplay between the host and the pathogen, highlighted by the activation of different cells by numerous cytokines and costimulatory molecules during different stages of JD pathogenesis. Our previous studies established the role of the TLR4 receptor in inflammation caused by MAP cell lysate immune challenge *in vitro*, and other studies also reported that polymorphisms in bovine *TLR4* were associated with increased MAP infection in dairy cattle (27, 28).

In this follow-up study, we explored the role of the TLR4 receptor in MAP infection using bovine MAC-T cells. The expression of WT and *TLR4*KO MAC-T cell miRNA and cytokine genes post-MAP infection was assessed, as were the concentrations of cytokines and chemokines in their culture supernatants. We found that the levels of CXCL10, TNF-α, IL-6, CXCL8, CCL2, and CCL3 were significantly induced in MAP-infected WT cells compared with uninfected WT cells. Conversely, significantly smaller amounts of IL-10 and VEGF-α were secreted by MAP-infected WT cells versus the uninfected cells. There were no significant differences between MAP-infected and uninfected WT cells for the remaining cytokines and chemokines (CCL4, IFN-γ, IL-36α, and IL-1α).

The observed decrease in IL-10 is inconsistent with the findings of other studies. For example, a study analyzed the transcriptome of bovine monocyte-derived macrophages infected with MAP and reported upregulated IL-10 expression 6 h postinfection compared to uninfected controls (29); this study, however, did not assess IL-10 expression at the protein level. Increased expression of IL-10 has been proposed to be a mechanism of immune evasion by MAP. It is possible that differences in cell type, MAP challenge level, and timing of sampling contributed to our contrasting results in terms of IL-10.

The higher concentration of IFN-γ in MAP-infected *TLR4*KO cells is consistent with the findings of another study that found higher concentrations of IFN-γ upon initial macrophage MAP infection (30). IL-10, CCL4, IL-36α, CCL2, and IL-1α were not significantly different between the MAP-infected and uninfected *TLR4*KO cells. This is inconsistent with findings that attribute activation of anti-inflammatory factors such as IL-10 as a survival tactic of MAP, as well as our previous work done with MAP-lysate (31, 32). The lowering of CXCL10 in edited MAC-T cells is consistent with the role of TLR4 in activating type 2 interferons, of which IFN-γ has a role in triggering the secretion of CXCL10 as a recruiter of CD4^+^ T-lymphocytes (33, 34). These results also indicate a possible role for TLR4 in CCL2 signaling during MAP infection, and the mechanism of activation may require live bacteria. Both CCL2 and CCL3 play roles in monocyte chemotaxis to sites of inflammation and are known to be markers of severe infection with Mycobacterium tuberculosis (35, 36). One explanation for these results could be that MAP induces CCL2 and CCL3 responses through TLR4, as the elimination of *TLR4* attenuated the response of CCL2 and CCL3 to MAP.

In terms of gene expression, we also observed that *TLR4*KO MAC-T cells displayed a higher proinflammatory cytokine gene response (*TNF-α*, *IL-1α*, *IL-1β*, and *IL-6*) when challenged with MAP. We attribute this higher proinflammatory cytokine expression in the KO cells to impaired TLR4 signaling. In contrast with these findings, our previous study reported that *TLR4*KO MAC-T cells were hyporesponsive to MAP-cell lysate (8). This opposite response could be attributed to different modes of interaction between cell lysate or live bacteria and the host cells. These results indicate that TLR4 may not be the only pattern recognition receptor responsible for the recognition of MAP and that knocking out *TLR4* in MAC-T cells impaired their responsiveness to MAP.

Toll-like receptors play an essential role in the host immune response to M. tuberculosis infection, especially TLR2 and TLR4, which lead to the activation of NF-κB signaling (37). Previous studies reported that by targeting TLRs miRNA can modulate the innate immune response against M. tuberculosis (38, 39). For example, miR-125a decreased macrophage inflammatory cytokine production during M. tuberculosis infection, and miR-125a levels were dramatically reduced by the knockdown of *TLR4* in THP-1 cells during M. tuberculosis infection (25). In contrast, the knockdown of *TLR2* had little effect on the expression of miR-125a, indicating that induction of miR-125a during M. tuberculosis infection is mainly dependent on TLR4 rather than TLR2 signaling. This could be one of the possible reasons for the stronger *TLR4*KO cell inflammatory response that was observed in the present study following MAP infection.

Limited studies have clarified the exact role of miRNA involved in host immune regulation in response to MAP infection in cattle. We found the expression of miR-133b, miR-84, and miR-92a was significantly induced in the WT cells post-MAP infection. However, there was no significant effect on their expression in *TLR4*KO cells. Notch signaling was reported to regulate innate immune responses by interaction with TLR signaling, especially for activation of macrophages (40), and Notch1 enhances inflammation of macrophages in response to TLR4 stimulation by altering NF-κB activation, and excessive interaction between Notch1 and TLR4 signaling may exacerbate the inflammatory response (41).

In this study, miR-133b and miR-184 were predicted to be involved in the Notch signaling pathway and their expression was not altered by MAP infection in *TLR4*KO cells, which implies that miR-184 may regulate the host inflammatory response by altering Notch/TLR4 interaction signaling. During MAP infection in cattle, miR-133b was reported to be involved in “lymphocyte activation” and miR-184 in “inflammatory response activation” (42).

In comparison, miR-92a negatively regulates the inflammatory response triggered by TLR signaling by directly targeting mitogen-activated protein kinase kinase 4 (MKK4) in LPS-infected macrophages. The MAP infection may regulate TLR signaling pathways and downstream immune responses by altering miRNA expression, and upregulating miR-146b in infected ileum may be one of the mechanisms by which MAP disrupts TLR signaling pathways after infection (42). In addition, a previous *in vivo* study also reported that the TLR signaling pathway was inhibited at 12 h post-MAP infection in the ileum (43). We found the expression of miRNAs increased significantly in WT cells after live MAP challenge, implying that those miRNAs may be involved in the molecular mechanisms regulating the host response to MAP.

Meanwhile, the expression of miRNAs in *TLR4*KO cells was not affected by MAP infection. Based on these results, we speculate that MAP infection may affect the TLR4 signaling pathway and downstream inflammatory responses by altering miRNA expression. Moreover, Liang et al. (42) reported that miR-146b is negatively correlated with the predicted target genes interleukin 4-receptor (IL4R) and spleen tyrosine kinase (SYK), which activates the NF-κB-mediated transcription of cytokines (44, 45), suggesting that miR-146b may suppress inflammatory responses triggered via the TLR signaling pathway. A previous study reported that miR-146a negatively regulates the inflammatory response induced by Porphyromonas gingivialis through TRAF6/p38 MAPK pathway (46). Overexpression of miR-133b was demonstrated to promote apoptosis of osteosarcoma cells by inhibiting the MAPK signaling pathway (47).

The phosphatidylinositol-3 kinases pathway, a critical pathway for host inflammatory response, target of the rapamycin (PI3K/Akt/mTOR), and TLRs/NF-ĸB signaling pathways cooperate in the regulation of the inflammatory response in mammary epithelial cells under Streptococcus uberis infection. In this study, we found that miR-133b may be involved in the inflammatory response induced by p38 mitogen-activated protein kinase (MAPK) signaling pathway by target gene function analysis. PI3 kinase pathway as one of the predicted pathways of miR-133b and miR-184 may contribute to host inflammation response to MAP challenge. As both pathway prediction tools demonstrated, miR-133b may also be involved in Wingless/Int1 (Wnt) signaling pathway which is highly interacted with numerous other signaling pathways, such as NF-κB, MAPK, protein kinase B (PKB/AKT), and signal transducer and activator of transcription (STAT) signaling (48).

In conclusion, *TLR4*KO MAC-T cells showed hyperresponsiveness to MAP infection compared to uninfected *TLR4*KO cells, which suggests that TLR4 is involved in modulating immune signaling and plays a pivotal role in determining the risk of MAP infection in epithelial cells. Also, these findings support that TLR4 may be an essential regulatory factor in the induction of miRNA (miR-199a, miR-92b, and miR-184) expression. Based on the above information, we hypothesize that TLR4 may be involved in MAC-T inflammatory response to live MAP and TLR4 expression levels may affect the host inflammatory response by regulating some signaling pathways.

## MATERIALS AND METHODS

### *TLR4* knockout cell line.

A *TLR4* knockout (*TLR4*-KO) cell line was previously developed in our laboratory using bovine mammary epithelial cells (MAC-T cells) by Shandilya et al. (8). The MAC-T cells were cultured in Dulbecco’s modified Eagle’s medium with 10% fetal bovine serum and penicillin-streptomycin (100 U/mL; Invitrogen; Thermo Fisher Scientific, Inc.), incubated at 37°C and 5% CO_2_, and cultured to a confluence of 80% as per Huynh et al. (49).

### MAP culture.

The MAP Madonna strain was gifted from the lab of Lucy Mutharia (University of Guelph, Guelph, Canada). The MAP was cultured in Middlebrook 7H9 broth (Sigma-Aldrich) supplemented with 10% oleic acid, albumin, dextrose, catalase (OADC; Becton, Dickinson, Canada), 0.05% Tween 80 (Sigma-Aldrich), and 2 mg/L mycobactin J (Allied Monitor Inc., Fayette, MO). The bacterial culture was incubated at 37°C and 5% CO_2_ as per our previous studies (50, 51).

### *In vitro* MAP challenge.

The *TLR4*KO and WT MAC-T cells were seeded at 1.2 × 10^5^ cells per well in separate 24-well plates and incubated overnight at 37°C and 5% CO_2_ to reach 80% confluence. Both cell types were either infected with MAP or provided an equivalent volume of MAP-carrier solution media (uninfected control) for 72 h. Bacterial CFU were determined using the pellet wet-weight method, whereby 1 mg of MAP Madonna pellet was equal to 10^7^ CFU (52). MAP was added to each cell type to achieve a 10:1 multiplicity of infection (53) and then spun for 2 min at 250 × *g* to ensure MAP interaction with the cells. This MAP challenge study was repeated in quadruplicate at independent times. The culture supernatants were collected and stored at −80°C until further analysis.

### Cytokines and chemokines multiplex analysis.

Analysis of culture supernatant cytokine and chemokine concentrations, namely, cytokines IFN-γ, IL-1α, IL-6, VEGF-α, TNF-α, IL-10, and IL-36-α, and chemokines CXCL8 (IL-8), CCL3 (macrophage inflammatory protein-1α [MIP-1α]), CCL4 (MIP-1β), and CXCL10 (IP-10) from MAP-infected and uninfected *TLR4*KO and WT MAC-T cells were outsourced to Eve technologies (Calgary, AB, Canada), where they were immunoassay multiplexed using the Luminex 100 system.

### RNA extraction.

Total RNA (mRNA + miRNA) extraction was carried out using the RNeasy minikit (Qiagen, Germany) from both uninfected (control) and MAP-infected samples of WT and *TLR4*KO MAC-T samples. The DNA traces were removed by DNase I treatment (Fermentas, Waltham, MA, USA) according to the manufacturer’s protocol.

### miRNA cDNA synthesis and quantitative PCR.

The miRNA cDNA synthesis was performed using Qiagen miRCURY LNA RT kit according to the manufacturer’s instructions, and cDNA samples were diluted to 1:20 with nuclease-free water. The quantitative PCR (qPCR) of previously identified candidate bovine miRNAs, namely, miR-92a, miR-184, and miR-133b from the literature (42, 54) was performed using a Step-One Plus qPCR machine (Applied Biosystem) using Sybr Green (Bio-Rad, Hercules, California). A master mix of 7 μL containing 5 μL 2× miRCURY SYBR Green Master Mix, 0.5 μL ROX Reference Dye, 1 μL resuspended PCR primer mix, and 0.5 μL RNase-free water was added in duplicate to 3 μL of diluted cDNA samples. Pooled samples were used to create a standard curve with a serial dilution of 1:5; this standard curve was used to ensure the efficiency of the reaction for all plates. The qPCR was subjected to denaturation at 95°C for 2 min, then to 40 PCR cycles of 95°C for 10 s, and then at 56°C for 1 min of primer annealing and amplification. The qPCR analysis was performed using the threshold cycle (ΔΔ*CT*) method with the change in miRNA expression being expressed as fold change (55), using miRNA U6 as the reference gene. All plates analyzed were required to have a standard curve with a reaction efficiency of 91 to 110%.

### mRNA cDNA synthesis and qPCR.

For mRNA cDNA synthesis, 500 ng of purified RNA was reverse transcribed to cDNA using the High-Capacity cDNA Reverse transcription kit (Applied Biosystems). For qPCR, the primer sequences for cytokine genes (Table 2) were selected from our previous study (8). Each qPCR was performed in duplicate in a total-reaction mixture of 10 μL comprising 2 μL of cDNA, 5 μL of 2× SYBR green master mix (ABI), 0.4 μL each of 10 pM forward and reverse primers, and 2 μL of nuclease-free water in a 96-well plate (ABI). The reactions were performed in a StepOne Plus instrument (ABI) using the following amplification conditions: 10 min at 95°C, 40 cycles of 15 s at 95°C (denaturation), and 1 min at 60°C (annealing + extension). The data were acquired using the ΔΔ*CT* method (55) and analyzed with two reference genes (*GAPDH* and *B2M*) as the internal controls.

**TABLE 2:** Details of primer sequences, annealing temperature, slope, and PCR efficiency for the target genes used for qPCR^*a*^

Gene	Primer sequence (5′–3′)	T_a_ (°C)	Slope	PCR efficiency (%)
*IL1α-F*	TTGGTGCACATGGCAAGTG	58.3	−3.260	103
*IL1α-R*	GCACAGTCAAGGCTATTTTTCCA			
*IL1β-F*	GCCTTCAATAACTGTGGAACCAAT	58.3	−3.161	107
*IL1β-R*	GTATATTTCAGGCTTGGTGAAAGGA			
*IL6-F*	GGCTCCCATGATTGTGGTAGTT	58.3	−3.360	98
*IL6-R*	GCCCAGTGGACAGGTTTCTG			
*TNF-α-F*	CGGTGGTGGGACTCGTATG	58.3	−3.125	109
*TNF-α-R*	CTGGTTGTCTTCCAGCTTCACA			
*SOCS3-F*	GCCACTCTCCAACATCTCTGT	58.3	−3.382	98
*SOCS3-R*	TCCAGGAACTCCCGAATGG			
*IL10-F*	AAAGCCATGAGTGAGTTTGACA	58.3	−3.380	98
*IL10-R*	TGGATTGGATTTCAGAGGTCTT			
*GAPDH-F*	TGGAAAGGCCATCACCATCT	60	−3.387	97
*GAPDH-R*	CCCACTTGATGTTGGCAG			
*B2M-F*	CTGCTATGTGTATGGGTTCC	60	−3.214	105
*B2M-R*	GGAGTGAACTCAGCGTG			

a F, forward; R, reverse. Ta, annealing temperature.

### Target gene prediction and pathway analysis.

The identification of miRNA-targeted genes was performed using two online tools, namely, TargetScan (http://www.targetscan.org) and mirDB (http://www.mirdb.org/index.html). Stringent selection criteria of target genes for both tools were applied; cumulative weighted context++ score less than −0.4 for TargetScan and target score >70 for miRDb (56, 57). Only the commonly identified target genes were considered for functional analysis. To facilitate the interpretation of gene targets and aid in the understanding of the potential function of the miRNAs, enrichment analysis for gene ontology (GO) annotation (molecular function, cellular component, and biological process) was performed using WebGestalt (http://www.webgestalt.org/#, version 2019).

### Statistical analysis.

To compare levels of different cytokines/chemokines, and mRNA and miRNA expression in MAP-infected treatments (MAP-infected versus uninfected controls), the values were analyzed using a two-way ANOVA test followed by the Bonferroni test (GraphPad Prism Software), and a *P* value of ≤0.05 was considered statistically significant. All data (*n* = 4) are presented as the mean ± standard error of the mean (SEM).

### Data availability.

The raw data sets from this study are available from the corresponding author upon reasonable request.
